# House officers’ specialist career choices and motivators for their choice– a sequential mixed-methods study from Malaysia

**DOI:** 10.1186/s12909-022-03845-2

**Published:** 2022-11-16

**Authors:** Anuradha Nadarajah, Pathiyil Ravi Shankar, Sivakumaran Jayaraman, Chandrashekhar T. Sreeramareddy

**Affiliations:** 1 School of Postgraduate Studies, International Medical University, Kuala Lumpur, Malaysia; 2 IMU Centre for Education, International Medical University, Kuala Lumpur, Malaysia; 3 Hospital Selayang, Ministry of Health, Kuala Lumpur, Malaysia; 4 Department of Community Medicine, International Medical University, Kuala Lumpur, Malaysia

**Keywords:** Career choic, Specialization, Motivation, Medicine, Delivery of healthcare, Perception

## Abstract

**Purpose:**

Shortage and maldistribution of medical specialists hamper healthcare quality. The specialist career choices of house officers determines the future composition of healthcare systems. We studied house officers’’ specialist career choices and motivators for their choice.

**Participants and methods:**

We conducted online in-depth interviews among seven house officers using an interview guide developed based on a literature review. The transcripts were analyzed. Major themes were identified. A 33-item questionnaire was developed, and the main and sub-themes were identified as motivators for specialist career choice. An online survey was done among 185 house officers. Content validation of motivators for specialist choice was done using exploratory factor analysis. First, second and third choices for a specialist career were identified. Multinomial logistic regression analyses were done to determine the socio-demographic factors and motivators associated with the first choice.

**Results:**

HOs perceived that specialist training opportunities provide a wide range of clinical competencies through well-structured, comprehensive training programs under existing specialist training pathways. Main challenges were limited local specialist training opportunities and hurdles for ‘on-contract’ HO to pursue specialist training. Motivators for first-choice specialty were related to ‘work schedule’, ‘patient care characteristics’, ‘specialty characteristics’, ‘personal factors’, ‘past work experience’, ‘training factors’, and ‘career prospects.’ House officers’ first choices were specialties related to medicine (40.5%), surgery (31.5%), primary care (14.6%), and acute care (13.5%). On multivariate analysis, “younger age”, “health professional in the family”, “work schedule and personal factors”, “career prospects” and “specialty characteristics” were associated with the first choice.

**Conclusions:**

Medical and surgical disciplines were the most preferred disciplines and their motivators varied by individual discipline. Overall work experiences and career prospects were the most important motivators for the first-choice specialty. The information about motivational factors is helpful to develop policies to encourage more doctors to choose specialties with a shortage of doctors and to provide career specialty guidance.

**Supplementary Information:**

The online version contains supplementary material available at 10.1186/s12909-022-03845-2.

## Background

Human resources for health (HRH) are a critical component of any healthcare system. The number and type of medical specialists must be aligned to the local disease burden. [1] and the availability of specialists is determined by training pathways and ‘brain drain’. [2] Shortage and maldistribution of specialists persist in Malaysia like in other developing countries.[3–5]. In Malaysia, house officers (HOs) undergo training in different specialties after graduation. The recent increase of enrollment into postgraduate training programs under the existing pathways [6] has led to the graduation of about 1000 new specialists in 30 disciplines annually. [5] Nevertheless, the maldistribution of specialists by private versus public sector, geographic distribution, and relative shortage of specialist doctors adversely affects the quality of care and hampers the training of junior doctors. [3, 7] 

Specialty choice of medical students, interns, and medical graduates is well researched globally [8, 9]. A complex decision-making process of specialty career choice [10] is influenced by personal, and professional circumstances, training opportunities, and prospects upon graduation [11–14]. The choice and factors driving the choice vary among students, interns, or house officers and by country. [8, 9] Survey research using ad hoc questionnaires often fails to obtain a deeper perspective about the reasons/motivators for respondents’ preferred specialty choice [8, 9]. For a complex decision-making process of specialist career choice, it is important to study why and how the decisions are made [10]. Qualitative studies provide deeper insights into the motivators for career specialty choices [15–17]. A recent critical review examined the role of different theories including the Myers-Briggs type indicators, Holland’s theory of vocational personalities, attachment theory, self-determination theory, social cognitive career theory and choice theory in specialty preferences among medical graduates [18]. Personality traits influence career interest, and along with motivation, and expected capability can influence the choice of a specialty. Achieving career-related, life-related, and overall quality of life can influence continued motivation and the willingness to maintain a career choice. External factors like parents, peers, social and cultural factors, values and beliefs, race and ethnicity can influence career choice.

Better understanding of motivators from both personal and professional domains can help provide career guidance to young graduates and inform policy decisions about specialist training needs. Existing Malaysian studies only used questionnaire surveys among medical students[11–14] and did not qualitatively explore what are the motivators for their specialist career choice. Perspectives of HOs who are at a transitional phase of their career are critical for health manpower policy decisions were not studied in Malaysia. In Malaysia, medical graduates do a two-year housemanship after completing the MBBS course like the internship in other British commonwealth countries. After completing their housemanship they could be appointed as medical officers eventually leading to a postgraduation training supported by the government. Several medical colleges have been started recently in the private sector and there is a glut of medical graduates and about a thousand graduates must wait nearly a year for a job opening and only about 45–50% of those completing housemanship will be offered a permanent position [19]. Most medical officers (MO) are appointed on a contractual basis and may not be eligible for government support during postgraduation. These factors may influence the career decisions of medical graduates.

A WHO report published in 2018 showed Malaysia had 1.5 doctors per 1000 population. [20] and for primary care physicians the per capita density in urban areas is 1.5 per 1000 population while it is only 1.1 per 1000 in rural areas. [21] The number of specialists is only four per 10,000 population while the recommended ratio by organizations like the OECD is 14. [22] There is also a concentration of doctors in the urban areas especially the Klang valley. Doctors are also leaving the government sector to work in private institutions affecting their availability to poorer sections of the population.

We aimed to identify the HOs’ medical specialty career choices and qualitatively explore the motivational factors related to the domains of personal, professional and career prospects.

## Methods

### Ethical approval and consent to participate

Ethical approval was obtained from International Medical University Joint Committee for Research and Ethics (Project ID No MSPH I/2020(03)) and from Medical Research Ethics Committee, Ministry of Health, Malaysia (NMRR ID: 20–1547-55,138 IIR). Informed consent was obtained from all participants.

### Design

A mixed-methods (qualitative followed by quantitative) study was conducted among the HOs working in Selangor state, Malaysia from July to December 2020. The findings from the qualitative study were used to design the questionnaire for the quantitative study.

### Qualitative component

In-depth interviews were used to explore HO perceptions about motivational factors for their choice of specialist training and specialist training opportunities. Interviews were conducted in English using online platforms till data saturation. A semi-structured interview guide was developed based on literature review and informal discussions among the researchers, and with medical graduates, and postgraduate students (Additional File 1). A pilot IDI was conducted with a participant from another tertiary hospital in Malaysia, who had just completed housemanship. The interviews were audio-recorded with consent. The interview sessions were conducted online after obtaining written informed consent. The researchers adopted an ontological paradigm of bounded realism, a constructivist epistemology and used an interpretivist methodology. The authors were medical doctors, and the first author is currently involved in choosing her specialty while the three senior authors have already chosen their specialties. They may be living through or had lived through the experiences suggested by the respondents. A phenomenological approach was followed, and we explored the lived experiences and opinions of the HOs through our own understanding. Each interview lasted for 25–35 min. By the seventh participant, researchers attempted to reach saturation as no new themes were emerging. Saturation is, however, a concept not well-defined and may be determined by evaluator proclamation after examining the adequacy and comprehensiveness of the results. [18]

The recorded interviews were transcribed verbatim by the first researcher (AN) using Microsoft Office 365 dictation-transcription tool and manual corrections were done. Each participants’ transcript was coded before proceeding to the next participant. To ensure rigor, member checking was done whereby the transcript was shared with the respective interviewee. One researcher (PRS) also read through the transcripts and agreed/disagreed with the themes and subthemes identified by AN. The reasons for their choice for specialist training were identified followed by the themes and subthemes/codes addressing their perceptions on specialist training opportunities (Additional File 1). NVIVO version 1.0 (2020) was used to aid the coding process and for data management. The thematic analyses approach was used after anonymizing the transcripts. Initial reading and repeated readings of the transcript were done to obtain a general understanding, comprehension, and familiarization before coding. Constant comparison of themes and refinement were done along with the ongoing analysis. The themes and subthemes and the results were also shared with the participants. Member checking enables the researchers to explore the extent to which their analysis and interpretations were accepted by the actual participants as reasonably representing their realities and experiences [23]. Study rigor was ensured by group involvement in developing the coding scheme, developing rules to arrive at mutually agreed codes, subthemes and themes, and all major decisions made during the study were documented in the student logbook. [24]

### Cross-sectional study design, setting, and participants

A cross-sectional online questionnaire survey was done among HO undergoing mandatory 2-years of clinical training for full registration as a medical officer. After one year of service as a medical officer, they are eligible for specialist training available in Malaysian public universities.

### Sample size and sampling

The sample size was calculated for a 95% confidence limit, 5% margin of error, and an anticipated proportion of 26%. [12] was 163 Expecting a non-response of 20% we adjusted our sample to 200 participants A list of HO was obtained from the Hospital director. From this list 364 HO 12 HO who were invited to participate in the interviews were excluded. Every second house officer was selected from an ordered frame of every K^th^ participant. N/n i.e., 352/200 ~ 2.

### Survey instrument

The questionnaire consisted of 3 parts: 1) sociodemographic, 2) intention to undergo specialist training and if intended then top three specialty choices, and 3) Motivators for Medical Specialist Career Choice. The motivators had 33 items derived from the raw data on major themes and sub-themes identified from IDI (Additional File 1). The motivators were measured on a five-point Likert Scale as the level of importance where 1 is ‘not important all’ and 5 is ‘very important.’ The questionnaire on motivators was reliable. Cronbach’s alpha value was 0.83 for the pre-test and 0.85 for the main survey. Exploratory factor analysis retained 26 out of 33 items and resulted in 7 constructs with 64.9% variance. Each construct demonstrated acceptable internal consistency. The seven constructs were labeled as “work schedule and personal factors”, “training factors”, “past work experiences”, “specialty characteristics”, “career prospects”, “patient care characteristics” and “social factors”. [25]

### Variables

Independent variables were sociodemographics and scores on motivators for the first-choice specialty (sum of all items under each domain (Additional File 1). Dependent variable was the first-choice specialty was grouped into four broad categories namely 1) medical, 2) surgical, 3) acute care and 3) primary care from the list of 23 specialties.

### Data collection procedure

An online questionnaire (via Google forms) was sent to consenting participants via e-mail or WhatsApp messenger. To improve the response rate, GrabFood vouchers worth Ringgit Malaysia RM 5 (about 1.25 USD) were offered as a token of appreciation for each completed survey, and friendly reminders were sent on the third, and the fifth day during the first week, and then weekly four weeks. A total of 185 completed questionnaires were received out of 262 participants without any incomplete items in the questionnaire.

### Data analyses

Data analyses were done using Statistical Package for Social Sciences (SPSS-IBM version 26). Normality of distribution for continuous data was tested. Continuous data for each item and domain scores of the motivators were computed. Cronbach’s alpha was estimated as a measure of internal consistency and a value greater than 0.7 indicates adequate internal consistency. To examine the test–retest reliability Pearson’s correlation coefficients and intraclass correlation coefficients (ICC) were estimated and a value greater than 0.75 indicated the stability or acceptable test–retest reliability. Exploratory factor analysis was done before inferential analyses. Items with factor loading 0.5 and above, items that form stable constructs (at least three items in one construct), and no cross-loading above 0.5 value were the criteria used to retain the items during the factor analysis. [26]

Descriptive statistics were used for demographics and specialist career choices. Multivariate analyses were done to assess the association of HO’s first choice of specialty with socio-demographic factors and the seven domain scores of motivators for the first choice. The factors that had a p-value less than 0.25 on univariate analyses were included in multivariate analyses to determine the factors associated with the first choice of specialty. As the first was a nominal variable (medical, surgical, acute care, and primary care), a multinomial regression analysis took surgical specialties as a ‘reference category’. Crude and adjusted odds ratios and their 95% confidence intervals (95% CI) were estimated. A p-value less than 0.05 was considered statistically significant.

## Results

### Qualitative findings

Seven (three males and four females) house officers aged 26—28 years who were in their fifth or sixth posting or currently in transition to being a medical officer were interviewed (Additional File 1). They were selected through purposive sampling and snow balling technic was also used to identify other HOs who may be interested. Two major themes that emerged were HO perceptions on specialist training in Malaysia” and “motivational factors for career specialty preferences”. The major theme of house officers’ perception of specialist training in Malaysia comprised two subthemes I. Advantages of specialist training in Malaysia and II. Perceived limitations and challenges of specialist training. The second major theme of motivational factors for their career specialty preference comprised seven subthemes: I. Work schedule, II. Patient care characteristics, III. Specialty characteristics IV. Personal factors, V. Past work experience, VI. Training factors, VII. Career prospects (Table 1).

**Table 1:** Subthemes and refined codes for motivational factors influencing first-choice specialty

Subthemes	Refined Codes
Work schedule	i. No on calls or less hectic on calls ii. Shift work iii. Fixed working hours
Patient care characteristics	i. Multidiscipline or wide variety of illness/cases ii. Acute management care of patient iii. Minimal interaction with patient iv. Quick results/recovery after intervention or treatment v. Continuous patient care
Specialty characteristics	i. Challenging nature of the field ii. Medical based iii. Surgical based iv. less medicolegal issues v. Involves more hands-on skill and procedures vi. Flexible working conditions vii. Prestige or reputation of the specialty
Personal factors	i. Family or relative influences/advice ii. Better work life balance iii. Personal interest in the specialty iv. Job satisfaction v. Medical school experiences vi. Social media or public figure influence
Past work experiences	i. Good teamwork in the department ii. Critical events or defining moment during HO rotations related to the specialty iii. Guidance and teaching activities in the department iv. Specialist or senior colleague role model/ influences
Training factors	i. Availability of parallel pathway ii. Availability of preparatory /training courses iii. Length of training iv. Cost of training
Career prospects	i. Future opportunities in private sector or private practice ii. Financially rewarding iii. Variety of subspecialties in the field to venture iv. Future teaching opportunities

## “House officers’ perception on specialist training in Malaysia”

### Advantages of specialist training opportunities in Malaysia

Recognition and availability of alternate/parallel pathway: The alternate or parallel pathway refers to specialization through a locally recognized overseas postgraduate program such as MRCP (Member of the Royal College of Physicians), MRCOG (Member of the Royal College of Obstetricians and Gynaecologists), and MRCPH (Member of the Royal College of Pediatrics and Child Health) where trainees work under supervision in accredited Ministry of Health or university hospitals.

A structured program and in-depth training: Few participants also mentioned that specialist training in Malaysia is well-structured, comprehensive, and offers in-depth training in different procedures. A participant mentioned.

*“The thing about our specialist training is because we have a lot of on job training, the Master's program students are exposed to a lot of procedures for example, in our hospitals, we can do a lot of hands-on.” (P 6)*

Mandatory HO training and rotation in various departments allows the junior doctors to have an overview of various specialties before pursuing specialist training.

### Perceived limitations and challenges of specialist training

Many participants mentioned limitation of intake in local training programs, contract doctors' challenges, lack of standardization of specialist training exam, nonavailability of recognized alternate pathways for certain specialties, lack of guidance and information regarding specialist training, longer duration, and lack of direct career pathway for some sub-specializations. Many young doctors are appointed on contract and these doctors are not eligible for government support during postgraduation.

“Tough times for contract doctors to apply for masters; even if manage to obtain a seat, the cost needs to be covered on own for now for masters, if no HLP [federal government scholarship] difficult” (P5).

“I think it's lacking clear information on specialist training because we don't have the idea yet of what we can do after our horsemanship.” (P2)

### Motivational factors for career specialty preferences

#### Work schedule

Most participants considered the working hours or timing of work by voicing their different preferences towards on calls, fixed working hours, or shift work.

“In radiology, I’ll have a fixed working hour with better work-life balance, I can go back at 5 pm, have public holidays off” (P6)

#### Patient care characteristics

Certain participants were interested in specialties that encounter the various type of patients; there is also a desire for a specialty that requires patient limited contact but contributes to the patient's diagnosis, such as radiology. Participants who favor surgical-based specialties indicated that they are attracted by quick results or patient improvement following the intervention.

#### Specialty characteristics

Challenging nature of the field, medically based, surgical based, less medico-legal problems, participation in hands-on skills and procedures, flexible working conditions, prestige, or credibility of the specialty were among the factors mentioned.

“Firstly, in the sense that I know I'm a surgical based person because I feel that I know even medical-based you can touch the patients, but I feel that surgical based it's more objective and then you can…I feel you arrive at a solution faster than a medical-based.” (P6).

#### Personal factors

Among those mentioned was family or relative influence, work-life balance, personal interest, job satisfaction, and social media or public figure influence. A respondent mentioned the influence of their teacher on their specialty preference.

#### Past work experiences

Past experiences in the specialty were identified as one of the motivational factors influencing the junior doctors’ preferred choice of specialist training.

“I graduated from ABC University and my surgeon back then was Dato DCE. He was a consultant in Hospital FGH at that point of time so I think the way he carried himself and his teaching made me like surgery since the beginning since I started my clinical years of medical school.”(P6).

Most of our participants highlighted that the availability of parallel pathways, availability of preparatory or training courses either locally or through distance learning in online mode, shorter length of training, and cost of training were all factors that they perceived would influence their.

#### Career prospects

The subtheme “career prospects” was influenced by future opportunities in the private sector or practice, financial rewards, job security, different sub-specialties in the field, and teaching opportunities.

“Then I think, especially when it comes to being a surgeon, the money that you get comes from the surgery itself, and surgery is quite expensive especially in the private division. That's why I think the financial stability there is a lot, a lot more than when you're like another regular practitioner or medical specialist. That's why [one of the reasons] I like becoming a surgeon” (P2).

The seven subthemes with the 33 refined codes for the major theme of motivational factors of career specialty preferences were utilized for developing the questionnaire.

### Quantitative results

The response rate was 71.1% (185/262). About two-thirds (63.8%) of the HOs were females, and the median age was 27 years (range 23–34 years). About 69% of them had never married and 30% were either married or into a relationship. More than half of them were Malays, 26% were Chinese, and 43% had obtained a graduate medical degree from Malaysian private medical schools (Table 2). The new instrument on motivators was validated by psychometric properties by exploratory analyses during which 26 items were retained of the initial 33 items accounting for a variance of 64.9%. [25]

**Table 2:** Socio demographics characteristics of the 185 HO who completed the questionnaire

	**Sociodemographic characteristics**	***N* = 185 (%)**
	***Age (in years)**** Median: 27 Mean: 26.7 SD: 1.6*	
Sex	Male	67 (36.2)
	Female	118 (63.8)
Age groups	23–26	91 (49.2)
	27–34	94 (50.8
Marital status	Single	128 (69.2)
	Married	29 (15.7)
	Into a relationship	27 (14.6)
	Divorced/Separated/Widowed	1 (0.5)
Ethnic origin	Malay	95 (51.4)
	Chinese	48 (25.9)
	Indian and others	42 (22.7)
Type of graduating university	Malaysian public university	39 (21.1)
	Overseas public university	37 (20.0)
	Malaysian private university	81 (43.8)
	Overseas private university	10 (5.4)

### Choice of specialist training

Broadly, the proportion of first-choice specialties related to medical, surgical, primary care, and acute care disciplines were 40.5%, 31.5%, 14.6%, and 13.5% respectively. The proportion of HO choosing medical specialties for second and third choice decreased to 35.7% and 26.5% respectively. Among the individual specialties, the ran order for the first choice was internal medicine (23.2%), general surgery (9.7%), emergency medicine (7.6%), public health (7.6%), and family medicine (7.0%). None of the HOs indicated otorhinolaryngology and nuclear medicine as their first choice (Table 3).

**Table 3:** HO choices for specialist training

Specialty	**First n (%)**	**Second n (%)**	**Third n (%)**
Internal Medicine	43 (23.2)	17 (9.2)	13 (7.0)
Pediatrics	7 (3.8)	11 (5.9)	3 (1.6)
Clinical Oncology	1 (0.5)	0(0)	4 (2.2)
Rehabilitation Medicine	1 (0.5)	5 (2.7)	4 (2.2)
Sports Medicine	5 (2.7)	7 (3.8)	4 (2.2)
Psychiatry	8(4.3)	13 (7.0)	7 (3.8)
Pathology	5 (2.7)	2 (1.1)	6 (3.2)
Radiology	4 (2.2)	4 (2.2)	4 (2.2)
Transfusion Medicine	1 (0.5)	4 (2.2)	4 (2.2)
Nuclear Medicine	0 (0)	2 (1.1)	0 (0)
**Medical Specialties**	**75 (40.5)**	**66 (35.7)**	**49(26.5)**
General Surgery	18 (9.7)	16 (8.6)	5 (2.7)
Orthopedics	6 (3.2)	9 (4.9)	13(7.0)
Neurosurgery	5 (2.7)	6 (3.2)	4 (2.2)
Otorhinolaryngology	0 (0)	8 (4.3)	4 (2.2)
Ophthalmology	12 (6.5)	2 (1.1)	7 (3.8)
Plastic Surgery	2 (1.1)	2 (1.1)	5 (2.7)
Pediatric surgery	1 (0.5)	11(5.9)	1 (0.5)
Forensic Medicine	2 (1.1)	2 (1.1)	6 (3.2)
Obstetrics and Gynecology	12 (6.5)	7 (3.8)	7 (3.8)
**Surgical specialties**	**58 (31.4)**	**52 (28.1)**	**52 (28.1)**
Emergency Medicine	14 (7.6)	15 (8.1)	5 (2.7)
Anesthesiology	11 (5.9)	10 (5.4)	6 (3.2)
**Acute care**	**25 (13.5)**	**25 (13.5)**	**11 (5.9)**
Public health/Community health	14 (7.6)	7 (3.8)	8 (4.3)
Family Medicine	13 (7.0)	23 (12.4)	17(9.2)
**Primary care**	**27 (14.6)**	**30 (16.2)**	**25 (13.5)**
No second or third choice	-	12 (6.5)	48(25.9)
**Total**	**185 (100)**	**185 (100)**	**185 (100)**

Bivariate analysis indicated that HO first-choice specialty was significantly different according to the sociodemographic factors such as age group (*p* = 0.046), marital status (*p* = 0.02), ethnicity (*p* = 0.015), having a medical professional in the family (*p* = 0.02), and graduation from a public or private medical school (*p* = 0.02). A higher proportion of single HOs chose medical, surgical, and acute care specialties compared to married HOs and those into a relationship. A higher proportion of Malay HO opted for acute care and primary care as the first choice than non-Malay HO. The proportion of acute care as the first choice was higher among HO who did not have a medical professional in the family. A higher proportion of graduates from private medical schools opted for medical, surgical, and acute care as their first choice (Table 4).

**Table 4:** Univariate analysis of sociodemographic factors and motivators associated with first-choice specialty of 185 HO **(**Reference dependent variable: Surgical specialties)

	Medical-based specialties *N* = 75			Acute care-related specialties *N* = 25			Primary care *N* = 27		
	Number (%)	Crude OR (95% CI)	p-value	Number (%)	Crude OR (95% CI)	p-value	Number (%)	Crude OR (95% CI)	*p*-value
**Age**									
< 27 (23 – 26)	37 (49.3)	1	0.288	13 (58.6)	1	0.577	7 (25.9)	1	0.006
≥ 27 (27 – 34)	38 (50.7)	1.46 (0.73, 2.91)		12 (41.4)	1.31 (0.51, 3.36)		20 (74.1)	4.05 (1.48, 11.08)	
**Sex**									
Male	27 (36.0)	1	0.406	8 (32.0)	1	0.345	7 (25.9)	1	0.132
Female	48 (64.0)	1.35 (0.6, 2.72)		17(68.0)	1.61 (0.60, 4.32)		20 (74.1)	2.17(0.79, 5.92)	
**Marital Status**									
Single	59 (78.7)	1	0.292	15 (60)	1	0.342	13 (48.1)	1	0.047
Married/into	16 (21.3)	0.65 (0.30, 1.44)		10 (40)	1.61 (0.60, 4.28)		14 (51.9)	2.60 (1.01, 6.67)	
**Ethnicity**									
Non-Malay	44 (58.7)	1	0.425	9 (36.0)	1	0.191	7 (7.8)	1	0.029
Malay	31 (41.3)	0.76 (0.38, 1.51)		16 (64.0)	1.91 (0.73, 5.00)		20 (74.1)	3.06(1.12, 8.35)	
**Has a health professional in the family or among relatives**									
Yes	36 (48.0)	1	0.670	4 (16)	1	0.004	12 (44.4)	1	0.533
No	39 (52.0)	1.61 (0.59, 2.31)		21 (84)	5.63 (1.72, 18.43)		15 (56.6)	1.34(0.54,3.35)	
**Public/private medical school**									
Private	51 (68.0)	1		15 (60.0)	1		9 (33.3)	1	
Public	24 (32.0)	0.67 (0.33, 1.36)	0.265	10 (40.0)	0.94 (0.36, 2.46)	0.907	18 (66.7)	2.83 (1.09, 7.37)	0.033
**Malaysian or overseas medical school**									
Overseas	25 (33.3)	1		8 (32.0)	1		13 (48.1)	1	
Local	50 (66.7)	0.97 (0.47, 2.02)	0.944	17 (68.0)	1.04 (0.38,2.82)	0.946	14 (51.9)	0.53 (0.21, 1.33)	0.175
**Stage of HO posting**									
4th—6th posting	35 (46.7)	1		12 (48)	1		12 (44.4)	1	
1st – 3rd posting	40 (53.3)	1.07 (0.54, 2.12)	0.854	13 (52)	1.01(0.40, 2.59)	0.982	15 (55.6)	1.17(0.47, 2.92)	0.742
**Domain scores of motivational factors (mean and SD)**									
Work schedule and personal	3.62 (0.71)	1.46 (0.92, 2.33)	0.109	3.53 (0.63)	1.23 (0.66, 2.29)	0.519	4.02 (0.63)	3.62 (1.75, 8.01)	0.001
Training related	3.96 (0.71)	1.35 (0.86, 2.13)	0.198	3.84 (0.61)	1.09 (0.59, 2.02)	0.780	4.13 (0.74)	1.90 (1.00, 3.63)	0.050
Past work experience	4.12 (0.74)	0.89 (0.55,1.44)	0.231	4.34 (0.63)	1.44 (0.70, 2.97)	0.320	4.18 (0.67)	0.998 (5.21,1.91)	0.996
Specialty characteristics	3.04 (0.79)	0.24 (0.14, 0.41)	< 0.001	3.72 (0.72)	0.660 (0.35, 1.23)	0.192	2.98 (0.91)	0.222 (0.12, 0.42)	< 0.001
Career prospects of the specialty	3.76 (0.85)	0.76 (0.50, 1.16)	0.204	3.44 (0.95)	0.494 (0.28, 0.87)	0.014^*^	4.06 (0.76)	1.21 (0.67, 2.18)	0.535
Patient care characteristics	3.56 (0.97)	0.96 (0.66, 1.41)	0.845	3.73 (0.78)	0.704 (0.70, 2.09)	0.487	3.37 (0.90)	0.473 (0.47, 1.27)	0.312
Social factors	2.86 (0.95)	0.68 (0.47, 0.99)	0.046	3.01 (0.73)	0.820 (0.49, 1.36)	0.445	2.70 (0.97)	0.56 (0.33, 0.94)	0.029

Among the motivators for first-choice specialty, mean scores for the domain of “work schedule and personal factors” (*p* = 0.005), “specialty characteristics” (*p* < 0.001), and “career prospects” (*p* = 0.029), showed significant differences according to first-choice specialty (Table 4). Primary care specialties had the highest mean score relative to other specialties for work schedule and personal factors, but it scored least (2.98) for the specialty characteristics. However, primary care (4.06) and surgical specialties (3.95) scored higher for career prospects.

### Multivariate analysis

#### Factors associated with the career specialty preference among house officers

The results of the two multinomial models were fitted as they fulfilled the model fitting criteria (*p* < 0.05) and goodness of fit (*p* > 0.05) are shown in Table 5. In model 1 age, “work schedule and personal factors”, “specialty /characteristics” and “career prospects” were associated with the first choice. HO aged 27 years and older (aOR 5.12, 95% CI 1.59,16.96, *p* < 0.05) had five times higher odds of choosing primary care than those aged below 27 years. HO who gave higher importance to “work schedule and personal factors” had higher odds of the first choice for specialties related to medicine (aOR 2.47, 95% CI 1.36,4.49, *p* < 0.05) and primary care (aOR 6.68, 95% CI 2.60,17.15, *p* < 0.001) compared to surgical specialties. HO who gave higher importance on “specialty characteristics” had lower odds for the first choice of specialties related to medicine (aOR 0.14, 95% CI 0.07,0.28, *p* < 0.001) and primary care (aOR 0.07, 95% CI 0.03,0.17, *p* < 0.001) compared to surgical specialties. HO who gave more importance to “career prospects” had a lower odds ratio for the first choice of specialties related to acute care (aOR 0.47, 95% CI 0.25,0.88) compared to surgical.

**Table 5:** Multinomial analyses sociodemographic factors and motivators associated with first-choice specialty of 185 HO (Reference dependent variable: Surgical specialties)

	**Medical** (*n* = 75)		**Acute care** (*n* = 58)		**Primary care (*n* = 27)**	
	Adj. OR (95% CI)	p-value	Adj. OR (95% CI)	*p*-value	Adj. OR (95% CI)	*p*-value
**Model 1**						
**Age (years)**						
< 27 (23 – 26)	1		1		1	
≥ 27 (27 – 34)	1.90 (0.83, 4.23)	0.128	1.33 (0.50, 3.56)	0.575	5.12 (1.59, 16.96)	0.006
**Domains of motivational factors**						
Work schedule and personal	2.47 (1.36, 4.49)	0.003	1.72 (0.86, 3.44)	0.127	6.68 (2.6, 17.15)	< 0.001
Specialty characteristics	0.14 (0.07, 0.28)	< 0.001	0.51 (0.24, 1.1)	0.086	0.07 (0.03, 0.17)	< 0.001
Career prospects of the specialty	1.03 (0.6, 1.74)	0.928	0.51 (0.28, 0.95)	0.034	1.51 (0.72, 3.12)	0.277
**Model 2**						
**Age (years)**						
< 27 (23 – 26)	1	0.122	1	0.814	1	0.012
≥ 27 (27 – 34)	1.94 (0.84, 4.47)		1.13 (0.41,3.13)		4.83 (1.41,16.57)	
**Has a health professional in the family or among the relatives**						
Yes	1	0.807	1	0.014	1	0.892
No	0.90 (0.39, 2.08)		4.73 (1.38, 16.3)		1.08 (0.34,3.48)	
**Undergraduate university**						
Public	1	0.258	1	0.788	1	0.093
Private	1.65 (0.69, 3.92)		0.87 (0.31, 2.45)		0.37 (0.12,1.18)	
**Domains of motivators**						
Work schedule and personal	2.51 (1.36, 4.62)	0.003	1.62 (0.80, 3.31)	0.183	6.66 (2.55,17.4)	< 0.001
Specialty characteristics	0.14 (0.07, 0.27)	< 0.001	0.52 (0.24, 1.14)	0.104	0.78 (0.03,0.19)	< 0.001
Career prospects of the specialty	1.00 (0.59, 1.71)	0.995	0.57 (0.30,1.07)	0.078	1.60 (0.75,3.40)	0.225

However, in model 2 when private or public medical school and health professional in the family was included age was not associated with the first choice of specialty, and among the motivators that were significant in model 1, “work schedule and personal factors” and “specialty characteristics” remained significant albeit with a small change in effect size but not the direction of the association. However, “career prospects” were not significant in model 2. HO without any health professionals in the family had higher odds for the first choice of specialties related to acute care (aOR 4.73, 95% CI 1.38,16.25, *p* < 0.05) compared to surgical specialties (Table 5).

## Discussion

Our study identified the major and sub-themes related to motivators for HOs first-choice specialist careers within the Malaysian context and informed the questionnaire about motivators that influence the first-choice specialty. HO reported that ‘work experiences’, ‘specialty characteristics’ and ‘work schedule’ related factors are considered in deciding on their first-choice specialty. A cross-sectional survey identified that first-choice specialties were medical, surgical, primary care, and acute care disciplines in that rank order. The first-choice specialty was associated with HO age, presence of health professionals in the family, and higher ratings of importance for motivators related to ‘work schedules’, ‘personal factors’, ‘specialty features’, and ‘career prospects.’

### Choice of specialist training

HOs first choice as medical disciplines were like the two previous studies among medical students from private medical schools in Malaysia. However, in clinical year students in a public medical school in Malaysia, surgery, and medicine disciplines were the top two choices [14]. Medical students are generally very familiar about these two disciplines hence they are very popular. In several studies from Asian countries, ‘internal medicine’ was reported as the first choice. [27–29] A study among first-year medical students in China, Sri Lanka, Nepal, India, and Malaysia reported that ‘surgery’ and ‘internal medicine’ were the top two choices [13]. The choices varied by region and countries globally [27]. For instance, in Gambia ‘internal medicine’ and ‘obstetrics and gynecology’ were the first choices for male and female medical students respectively [30]. However, in 11 Latin American countries, medical student’s top three choices were, ‘general surgery’, ‘pediatrics’, and internal medicine [31]; in six sub-Saharan African countries they were ‘general surgery’, ‘internal medicine’, and ‘pediatrics’ [32]. In New Zealand, the most popular career choice among junior doctors was medicine followed by ‘surgery’ and ‘general practice’. [33] The results are not comparable across the countries owing to a lack of representative samples and survey participants ranging from preclinical, clinical students, interns, and medical officers. [30–33] Decisions on specialist career choice is a complex process and likely to change as students’ progress through the medical training course as well as based on prevailing opportunities and prospects after graduation. [10]

In line with global trends, more HOs were female in our study. Data on the gender ratio of medical students is not available in Malaysia. Women doctors tend to concentrate in specialties related to providing care to the family and which allow for flexible working hours. [34] Society still expects females to be primarily responsible for child rearing and raising families and women doctors are more likely to choose part-time work. [35]

Recently several medical colleges have been opened in the country. A study in Nepal had shown that students who had received a government scholarship were more likely to work in Nepal and serve in rural areas. [36] Students from private medical colleges showed a greater intention to migrate to developed nations. In Malaysia the job satisfaction of workers in the public sector was lower than in the private sector and doctors mentioned pressure from administrative tasks and believed part of their work did not make sense. [37] Compared to the private sector, government doctors see a greater number of patients, have a greater workload, and are paid less. The excessively long working hours of junior doctors and the unfair contract system has also received scrutiny. [38]

Primary care disciplines were ranked third by HOs in our study, and in previous studies, these disciplines were also least preferred. [13] Improved primary care facilities in Malaysia and emphasis on public health and preventive care particularly during the Covid-19 pandemic during the survey are likely to have driven the HOs to rank these disciplines higher. Acute care disciplines were least preferred by the HO. Lack of options for sub-specialization, fast-paced and stressful work environment of acute care disciplines compared to disciplines related to medicine and surgery are likely reasons for these disciplines being least preferred. Disciplines such as oncology, nuclear medicine, rehabilitation, transfusion medicine, and otolaryngology were also the least preferred disciplines. A study from Saudi Arabia reported that these specialties were least preferred by young graduates*.*[39] Population aging and rising burden of chronic diseases are likely to increase the need for specialists in these disciplines. [40, 41]

HO aged > 27 years (compared to ≤ 27 years) choose primary care disciplines as the first choice, a decision likely be driven by better work-life balance offered by fixed working hours of primary care medicine. HO without health personnel in the family had higher odds of choosing acute care disciplines. Health personnel within a family circle would bring negative influence about the fast-paced, extended hours and stressful work of acute care disciplines. On a positive note, they may also help individuals formulate a clearer picture into the unfavourable working conditions and/or work-life balance challenges.

All the seven domains of motivators identified by the qualitative study scored a mean of over three (maximum score five) in a cross-sectional survey. HO ranked ‘work experience’ as the most important factor while making the final choice in a Malaysian survey of working doctors, [14, 42] and a qualitative study among postgraduate students in the UK. [43] Our results suggest that HOs are influenced by their conducive work environment and supportive supervision provided by specialists during their clinical rotations. Role-modelling by senior doctors and scaffolding are important factors in career choice. In the United Kingdom, a survey among medical students showed high quality general practice attachments can promote careers in general practice. [44] This can be an area for further research in the Malaysian context. The questionnaire on motivators was derived from IDI and the results of the survey confirm the qualitative results.

HOs who gave higher importance to “work schedule and personal factors” had higher odds of primary care or medicine disciplines as their first-choice specialty. In a study from the UK, “working hours and conditions” were important considerations to specialize as a ‘general practitioner’. [45] Improving work conditions, offering more flexibility and part-time work schedules, and improving knowledge about new and emerging specialties among HOs can be considered. These findings indicate that disciplines such as primary care, and public health that have fixed and flexible working hours are chosen as the first choice to maintain a better work-life balance. HOs who rated higher importance for ‘specialty characteristic’ had higher odds of choosing surgical disciplines as a first-choice specialty. The attributes of surgical disciplines such as skilled hands-on procedures, challenging nature of patients needing surgical interventions, and monetary rewards for surgeons in the private sector are likely to have influenced the HOs to rate higher on ‘specialty characteristics.

## Strengths and limitations

The main strength of this study was the qualitative exploration of motivators, preceding the survey to develop the questionnaire on motivators. The IDI followed quality checks as per the guidelines. To-date questionnaire studies have reported results on motivators for such detailed list of motivators using a validated research tool developed the authors. Future studies could adapt the instrument according to their local context. Mixed methods study provided an opportunity to verify the motivators of medical specialist career choice. The career choices of the HO from a single hospital limits generalizability of our findings to the entire nation. Nevertheless, qualitative findings provide a good reflection of drivers that influence the medical specialist career choice. The choices for specialist training are dynamic and likely to change. Non-response bias may have affected our results, perhaps non-responders would likely be those without any intention to pursue specialist training.

## Implications

HO choice of specialization and motivators influencing their choice using a robust research method can assist medical specialist workforce planning in Malaysia. Career guidance and improved hands-on experience during clinical postings in less preferred disciplines like oncology, rehabilitation medicine, and nuclear medicine, may be considered. Medical curricula should offer rotations in these disciplines to assist students understand their significance in future clinical practice. Improved training opportunities with multiple career pathways and financial incentives for less preferred specialties would influence HOs to choose them. As career intentions are likely to change, prospective study among the HO would help assess the intended choices and a final choice for a specialist career. A nationwide survey of HO is needed to better understand the career intentions of young medical graduates in Malaysia.

## Conclusion

Specialties related to medical and surgical disciplines were the most preferred for future training by HO. Planning including human resources for health, addressing brain drain and maldistribution are prevalent challenges in developing countries. Motivators for the first choice varied by discipline. Overall work experiences and career prospects were the most important motivators for the first-choice specialty. The information about motivational factors is helpful to develop policies to encourage more doctors to choose specialties with a shortage of doctors and to provide career specialty guidance.

## Supplementary Information

**Additional file 1.** 

**Additional file 2.**

## Data Availability

The data collected is available from the corresponding author upon reasonable request.

## Abbreviations

HRH: Human resources for health
HOs: House officers
IDI: In-depth interviews
MMSCCQ: Motivators for Medical Specialist Career Choice Questionnaire
RM: Ringgit Malaysia
ICC: Intraclass correlation coefficients
CI: Confidence interval
MRCP: Member of the Royal College of Physicians
MRCOG: Member of the Royal College of Obstetricians and Gynaecologists
MRCPH: Member of the Royal College of Pediatrics and Child Health
OR: Odds ratio
AOR: Adjusted odds ratio
