# Supplementary material for: House officers’ specialist career choices and motivators for their choice– a sequential mixed-methods study from Malaysia
Source: BMC Med Educ. 2022 Nov 16;22:796. doi: 10.1186/s12909-022-03845-2 (PMC9668396; doi:10.1186/s12909-022-03845-2)
Supplement: Supplementary file 1 — Additional file 1. [file 12909_2022_3845_MOESM1_ESM.docx]

**Appendix A**

**INTERVIEW GUIDELINE** (for interviewer use)

1. Basic Information

Date :

Time :

Interviewer :

Interviewee :

Posting : 5^th^ / 6^th^

Code for Data entry :

Contact detail :

**2. Introduction:**

- Introduction Ice breaking : Name, brief background, role in the study
- Purpose of the interview: To explore the perception on specialist training opportunities and to explore in depth the reasons associated with the choice among house officer
- Brief about the Interview (as in study information sheet): will be recorded, duration 30-40min,
- Confirmation on the informed consent, and reassure the confidentiality

**2. Opening question**:

Ice breaking questions. How are you? Are you working today Before starting interview, do you have concern or questions to ask regarding the study?

**3. Content Questions**

i) What is your opinion on specialist training opportunities in Malaysia?

*Probe:* What are the plus points?

*Probe:* What are the minus points?

ii) Do plan to specialize in future?

if Qii) answer No 🡪 *Probe*: you haven’t decided yet or have decided not to specialize?

if still decided not to specialize

*Probe:* Do you mind sharing the reasons why you chose not to specialize?

if haven’t decided

*Probe:* what is your preference of choice of specialty/specialist training?

*Probe:* which posting interested you the most and why?

if Q ii) answer Yes proceed to Q iii)

iii) What is your first 3 preferred choice of specialist training?

iv) What are the reasons/factors you considered for preferred choice of specialist training?

*Probe:* Any defining moment/ critical event that helped you make your

decision

*Probe:* Any experience that helped you make your decision?

*Probe:*  Any role model? – family, senior doctor

*Probe:* Did you have any medical school experiences that may have influenced your decision?

*Probe:* How about the timing of the work and work-life balance?

*Probe:* Any personal/ family reasons?

*Probe:* How are important are the nature/scope of job, job satisfaction, glamour, indemnity risk, potential to earn more money while choosing your specialty?

**4. Closing**

i) Any concern or questions to ask regarding the study?

ii) Thank you for the participation

iii) Request to follow up if any further queries or clarification about the answers

**APPENDIX B**

**Raw data on item pool generation for the “motivational factors of career specialty preference’ scale items in questionnaire:**

| **THEME 2 : MOTIVATIONAL FACTORS FOR CAREER SPECIALTY PREFERENCE** | | | | | |
| --- | --- | --- | --- | --- | --- |
| Open Coding/Free codes identified from transcripts  (managed in NVivo ) | Refined codes with grouping | Refinement of codes with revised grouping under main themes | Initial subthemes | **33 ITEMS GENERATED FROM REFINED CODES AND QUOTATION**  **(33 scale items)** | **REVISED TERMINOLOGY FOR SUBTHEMES / QUESTIONNAIRE DOMAIN**  **(7domains)** |
| - no on calls - flexible post call off - reasonable on calls + Less hectic on calls - Shift work - Fixed working hours - multidiscipline or variety of illness/cases handled - Short term management and no long term commitment with patient? Rephrase to acute - Frontline nature of work – rephrased acute - Minimal interaction with patient - Continuous patient care - Challenging nature of the field - Quick results after intervention or treatment - Job content- comprised in specialty characteristics - medical based - surgical based - Less medicolegal issues - Involve hands on skill or experience - No ward rounds -omitted-cause similar like minimal interaction with patient? - Flexible working - Prestige - Family or relative influences/advice - Better work life balance - Personal interest - Interest in Urgent care- omit/ changed (refined acute care) - Job satisfaction - Medical school experiences - Social media or public figure influence - Good teamwork in the department - Critical events and defining moment - Guidance and teaching activities in the department - Working with skillful staffs in the department -combine as teamwork - Work like family – combine with team work - Specialist approachable and teach - specialist influence /guidance and teaching activities in department - Specialist or senior colleague’s influences/role model - HO posting experiences – renamed events/defining moment during HO - Personal or pleasant working experience🡪 - availability of recognized alternate / parallel pathway - Availability of preparatory programs locally - Availability of distance learning/ online training programs - Length of training(shorter training period) - Less expensive cost of training - Future opportunities in private sector - Private practice – combine with private sector - Financial reward - Variety of subspecialties in the - Can conduct courses – teaching opportunities | no on calls  flexible post call off  reasonable on calls  Less hectic on calls  (On call) | - No or less hectic on calls - Shift work - Fixed working hours   (timing of work) | Timing of work | **A1 No or less hectic on calls**  **A2 Shift work**  **A3 Fixed working hours** | **A WORK SCHEDULE** |
|  | Shift work  Fixed working hours  ( working hour) |  |  |  |  |
|  | - multidiscipline or wide variety of illness/cases handled - Acute management of patient - Minimal interaction with patient - Continuous patient care   (nature of patient care) | - multidiscipline or wide variety of illness/cases handled - Acute management of patient - Minimal interaction with patient - Continuous patient care - Quick results/recovery after intervention or treatment common after recovery   (nature of patient care) | Nature of patient care | **B4 Multidiscipline or wide variety of cases handled**  **B5 Acute patient care**  **B6 Minimal interaction with patient**  **B7 Continuous patient care**  **B8 Quick results/recovery after intervention** | **B PATIENT CARE CHARACTERISTICS** |
|  | - Challenging nature of the field - medical based - surgical based - Less medicolegal issues - Involve hands on skill or experience - Flexible working conditions - Prestige/reputation of the specialty - **Quick results/recovery after intervention or treatment (shift to theme 2)**   **(job content/ characteristics)** | - Challenging nature of the field - medical based - surgical based - Less medicolegal issues - Involve hands on skill or experience - Flexible working conditions - Prestige/reputation of the specialty   (Specialty characteristics) | Specialty characteristics | **C9 Challenging nature of the field**  **C10 Medical based**  **C11 Surgical based**  **C12 Less medicolegal issues**  **C13 Involves more hands-on skill or experience**  **C14 Flexible working conditions**  **C15Prestige/reputation of the specialty** | **C SPECIALTY**  **CHARACTERISTICS** |
|  | - Family or relative influences/advice - Better work life balance - Personal interest - Job satisfaction - Medical school experiences - Social media or public figure influence   ( Personal factors/ reasons) | - Family or relative influences/advice - Better work life balance - Personal interest - Job satisfaction - Medical school experiences - Social media or public figure influence     (Personal factors/ reasons) | Personal factors/ reasons | **D16 Family or relative influences/advice**  **D17 Better work life balance**  **D18 Personal interest**  **D19 Job satisfaction**  **D20 Medical school experiences**  **D21 Social media or public figure influence** | **D PERSONAL FACTORS** |
|  | - Good teamwork in the department - Events/ defining moment during HO rotations - Guidance and teaching activities in the department - Specialist or senior colleagues influences/role model   **(Working experiences in the department)** | - Good teamwork in the department - Events/ defining moment during HO rotations - Guidance and teaching activities in the department - Specialist or senior colleagues influences/role model   **(Working experiences/condition in the department)** | (Working experiences/condition in the department) | **E22 Good teamwork in the department**  **E23 HO posting experiences**  **E24 Guidance and teaching activities in the department**  **E25 Specialist or senior colleagues influences/role model** | **E PAST WORKING EXPERIENCE** |
|  | - availability of recognized alternate / parallel pathway - **Availability of preparatory/training programs locally** - **Availability of distance learning/ online training/preparatory courses 🡪 combine with above.** - Length of training (shorter training period) - cost of training (less expensive)   **(specialist training courses/education factors)** | - availability of recognized alternate / parallel pathway - Availability of preparatory/training programs locally - Availability of distance learning/ online training/preparatory courses 🡪 combine with above.   🡪Length of training (shorter training period)  🡪cost of training (less expensive)  (specialist training courses/education factors) | specialist training courses/education factors | **F26 Availability of parallel pathway**  **F27 Availability of preparatory/training**  **Courses**  **F28Length of training (shorter training period)**  **F29 cost of training (less expensive)** | **F TRAINING FACTORS** |
|  | - Future opportunities in private sector or practice - Financially rewarding - Various subspecialties in the field to venture - Future teaching opportunities   (Prospect of career in the specialty) | - Future opportunities in private sector or practice - Financially rewarding - Various subspecialties in the field to venture - Future teaching opportunities   (Prospect of career in the specialty) | - **Prospect of career in the specialty)** | **G30 Future opportunities in private sector or practice**  **G31 Financially rewarding**  **G32 Various subspecialties to venture**  **G33 Future teaching opportunities** | **G CAREER PROSPECTS** |

**Appendix C**

**QUESTIONNAIRE**

**PART I**

Please **do not** state your name on the questionnaire.

This section contains questions on **sociodemographic details.** Kindly tick on one relevant answer for each question except for question no 9 where you may choose more than 1 answer and kindly specify the details for the relevant questions as mentioned.

1. Gender: ☐ Male ☐ Female

2. Age: ___

3. Marital Status: ☐ Single ☐ Married ☐ Into a relationship ☐ Separated/Widowed/Divorced

4. No of Children: ____

5. Ethnicity : ☐ Malay ☐ Chinese ☐ Indian ☐ Others , specify:….........

6. Undergraduate University: ☐ Malaysian Public University

☐ Private Medical University in Malaysia (all years locally)

☐ Private Medical University in Malaysia (twinning program: clinical years in overseas )

☐ Overseas Public University

☐ Overseas Private University

7. Current HO posting : ☐ 1^st^ ☐ 2^nd^ ☐ 3^rd^ ☐ 4^th^ ☐ 5^th^ ☐ 6^th^

8. Current HO department:

1. Accident and Emergency Department
2. Anesthesiology Department
3. Internal Medicine
4. Obstetrics and Gynaecology Department
5. Orthopedic Department
6. Pediatrics Department
7. Surgical Department
8. Psychiatry

9. Any of your immediate family member or close relatives is working in health profession?

(You may choose more than 1 answer for this question)

☐ Father

☐ Mother

☐ Siblings

☐ Spouse

☐ Other close relatives in family circle

☐ None/ Not Applicable

10. Based on Q9; please **specify their medical profession and if doctor please specify their specialty**

E.g : Father : surgeon , Spouse : medical officer , Aunty : staff nurse

specify___________

**PART II**

This section consists of details on **choice of specialist training**. Choose **only ONE answer for each question.**

.

11 What is your **first (1^st^)** preferred choice of specialist training. Choose **one (1)** answer

(as drop list in Google form)

☐ Ophthalmology

☐ Obstetrics and Gynecology

☐ Psychiatry

☐ Pathology

☐ Public Health/ Community

☐ Plastic Surgery

☐ Pediatrics

☐ Rehabilitation Medicine

☐ Radiology

☐ Sports Medicine

☐ Transfusion Medicine

☐ Anesthesiology

☐ Clinical Oncology

☐ Emergency Medicine

☐ Family Medicine

☐ Forensic Medicine

☐ General Surgery

☐ Internal Medicine

☐ Nuclear Medicine

☐ Neurosurgery

☐ Otorhinolaryngology (ORL/ENT)

☐ Orthopedic

12. What is your **second (2^nd^ )** preferred choice of specialist training Choose **one (1)**answer

Same list provided as first choice with add on :

24) ☐ I don’t have a second choice

13. What is your **third (3^rd^ )** preferred choice of specialist training .Choose **one (1)**answer

Same list provided as first choice with add on;

25) ☐ I don’t have a third choice

**PART III: Motivating factors for career specialty preference**

Indicate to what extent the following factors are important for you in choosing your preferred choice of specialty.

For each item, please select the appropriate answer according to the importance scale/score between 1 to 5 as indicated below:

1: Not important at all

2: Not important

3: Somewhat important

4: Important

5: Very important

This section is based on reasons or factors that motivate you to choose your most preferred specialty (1st choice) to specialize in future.

*if you are using mobile phone to answer, you might need to slide left for scale 5.

| **Motivational Factors / Reasons for the preferred choice of specialty** | | | | | | |
| --- | --- | --- | --- | --- | --- | --- |
| **A WORK SCHEDULE** | | **1** | **2** | **3** | **4** | **5** |
| 1 | No on calls or less hectic on calls |  |  |  |  |  |
| 2 | Shift work |  |  |  |  |  |
| 3 | Fixed working hours |  |  |  |  |  |
| **B PATIENT CARE CHARACTERISTICS** | | **1** | **2** | **3** | **4** | **5** |
| 4 | Multidiscipline or wide variety of illness/cases |  |  |  |  |  |
| 5 | Acute management care of patient |  |  |  |  |  |
| 6 | Minimal interaction with patient |  |  |  |  |  |
| 7 | Quick results/recovery after intervention or treatment is common in this field |  |  |  |  |  |
| 8 | Continuous patient care |  |  |  |  |  |
| **C SPECIALTY CHARACTERISTICS** | | **1** | **2** | **3** | **4** | **5** |
| 9 | Challenging nature of the field |  |  |  |  |  |
| 10 | Medical based |  |  |  |  |  |
| 11 | Surgical based |  |  |  |  |  |
| 12 | Less medicolegal issues |  |  |  |  |  |
| 13. | Involves more hands-on skill and procedures |  |  |  |  |  |
| 14. | Flexible working conditions |  |  |  |  |  |
| 15. | Prestige or reputation of the specialty |  |  |  |  |  |
| **D PERSONAL REASON /FACTOR** | | **1** | **2** | **3** | **4** | **5** |
| 16. | Family or relative influences/advice |  |  |  |  |  |
| 17. | Better work life balance |  |  |  |  |  |
| 18. | Personal interest |  |  |  |  |  |
| 19. | Job satisfaction |  |  |  |  |  |
| 20. | Medical school experiences |  |  |  |  |  |
| 21. | Social media or public figure influence |  |  |  |  |  |
| **E**  **PAST WORK EXPERIENCE** | | **1** | **2** | **3** | **4** | **5** |
| 22. | Good teamwork in the department |  |  |  |  |  |
| 23. | Critical events or defining moment during HO rotations related to the specialty |  |  |  |  |  |
| 24. | Guidance and teaching activities in the department |  |  |  |  |  |
| 25 | Specialist or senior colleague role model/ influences |  |  |  |  |  |
| **F TRAINING RELATED FACTORS** | | **1** | **2** | **3** | **4** | **5** |
| 26. | Availability of parallel pathway |  |  |  |  |  |
| 27. | Availability of preparatory /training courses either locally or distance learning/ online mode |  |  |  |  |  |
| 28. | Length of training (shorter training period) |  |  |  |  |  |
| 29. | Cost of training (less expensive) |  |  |  |  |  |
| **G CAREER PROSPECTS** | |  |  |  |  |  |
| 30. | Future opportunities in private sector or private practice |  |  |  |  |  |
| 31. | Financially rewarding |  |  |  |  |  |
| 32. | Variety of subspecialties in the field to venture |  |  |  |  |  |
| 33. | Future teaching opportunities |  |  |  |  |  |

-------------------------------------------THANK YOU---------------------------------------------------

**Appendix D**

**Table Sociodemographic characteristics of in-depth interview participants**

| Characteristics | Participants  (n) | Characteristics | Participants  (n) |
| --- | --- | --- | --- |
| *Gender*  Male  Female  *Age*  26  27  28  *Marital status*  Single  Married | 3  4  1  3  3  6  1 | ***Ethnicity***  Malay  Indian  Others  ***Specialty of first preference***  Accident and Emergency  Anaesthesiology  Internal Medicine  Orthopaedics  Psychiatry  Radiology  Surgery | 3  3  1  1  1  1  1  1  1  1 |
