# Supplementary material for: House officers’ specialist career choices and motivators for their choice– a sequential mixed-methods study from Malaysia
Source: BMC Med Educ. 2022 Nov 16;22:796. doi: 10.1186/s12909-022-03845-2 (PMC9668396; doi:10.1186/s12909-022-03845-2)
Supplement: Supplementary file 2 — Additional file 2. [file 12909_2022_3845_MOESM2_ESM.docx]

1. COREQ (COnsolidated criteria for REporting Qualitative research) Checklist

| **Topic** | **Item No.** | **Guide Questions/Description** | **Reported on**  **Page No.** |
| --- | --- | --- | --- |
| **Domain 1: Research team**  **and reﬂexivity** | | | |
| *Personal characteristics* | | | |
| Interviewer/facilitator | 1 | Which author/s conducted the interview or focus group? | The first author (AN) |
| Credentials | 2 | What were the researcher’s credentials? E.g. PhD, MD | The researcher is a medical doctor (MBBS), MPH student with additional training in qualitative research |
| Occupation | 3 | What was their occupation at the time of the study? |  |
| Gender | 4 | Was the researcher male or female? | Female |
| Experience and training | 5 | What experience or training did the researcher have? | The researcher had additional training in qualitative research |
| *Relationship with*  *participants* | | | |
| Relationship established | 6 | Was a relationship established prior to study commencement? | The interviewer contacted the participants online before starting the study. |
| Participant knowledge of  the interviewer | 7 | What did the participants know about the researcher? e.g. personal goals, reasons for doing the research | The researcher knew some of the participants. Detailed information about the research was provided. |
| Interviewer characteristics | 8 | What characteristics were reported about the inter viewer/facilitator?  e.g. Bias, assumptions, reasons and interests in the research topic | The interviewer was a medical doctor and a student of public health. She is also interested in securing a suitable postgraduate position. She reflected on her biases and world view and the possible impact it would have on her interpretation before and after the interview and discussed these with her supervisors |
| **Domain 2: Study design** | | | |
| *Theoretical framework* | | | |
| Methodological orientation and Theory | 9 | What methodological orientation was stated to underpin the study? e.g. grounded theory, discourse analysis, ethnography, phenomenology, content analysis |  |
|  | | | |
| Sampling | 10 | How were participants selected? e.g. purposive, convenience, consecutive, snowball | Purposive sampling and snowballing |
| Method of approach | 11 | How were participants approached? e.g. face-to-face, telephone, mail, email |  |
|  |  |  | Through email and WhatsApp messenger |
| Sample size | 12 | How many participants were in the study? | Seven |
| Non-participation | 13 | How many people refused to participate or dropped out? Reasons? | Three. No reasons were provided. |
| *Setting* | | | |
| Setting of data collection | 14 | Where was the data collected? e.g. home, clinic, workplace | The data were collected online |
| Presence of non-  participants | 15 | Was anyone else present besides the participants and researchers? | No one else was present. |
| Description of sample | 16 | What are the important characteristics of the sample? e.g. demographic  data, date | These have been described in the manuscript. |
| *Data collection* | | | |
| Interview guide | 17 | Were questions, prompts, guides provided by the authors? Was it pilot  tested? | Yes an interview guide was developed and it was pilot-tested. |
| Repeat interviews | 18 | Were repeat interviews carried out? If yes, how many? | No repeat interviews were done. |
| Audio/visual recording | 19 | Did the research use audio or visual recording to collect the data? | The interviews were audio-recorded. |
| Field notes | 20 | Were ﬁeld notes made during and/or after the inter view or focus group? | Notes were made during the interview and finalized immediately after. |
| Duration | 21 | What was the duration of the inter views or focus group? | Approx 30 minutes. |
| Data saturation | 22 | Was data saturation discussed? | Yes |
| Transcripts returned | 23 | Were transcripts returned to participants for comment and/or  correction? | Yes |
| **Domain 3: analysis and**  **ﬁndings** | | | |
| *Data analysis* | | | |
| Number of data coders | 24 | How many data coders coded the data? | Two |
| Description of the coding  tree | 25 | Did authors provide a description of the coding tree? | Yes |
| Derivation of themes | 26 | Were themes identiﬁed in advance or derived from the data? | A mixture of inductive and deductive approaches was followed. |
| Software | 27 | What software, if applicable, was used to manage the data? | NVIVO |
| Participant checking | 28 | Did participants provide feedback on the ﬁndings? | Yes |
| *Reporting* | | | |
| Quotations presented | 29 | Were participant quotations presented to illustrate the themes/ﬁndings?  Was each quotation identiﬁed? e.g. participant number | Yes |
| Data and ﬁndings consistent | 30 | Was there consistency between the data presented and the ﬁndings? | Yes |
| Clarity of major themes | 31 | Were major themes clearly presented in the ﬁndings? | Yes |
| Clarity of minor themes | 32 | Is there a description of diverse cases or discussion of minor themes? | Yes |

Developed from: Tong A, Sainsbury P, Craig J. Consolidated criteria for reporting qualitative research (COREQ): a 32-item checklist for interviews and focus groups. *International Journal for Quality in Health Care*. 2007. Volume 19, Number 6: pp. 349 – 357
